# The Secondary Motor Cortex-External Globus Pallidus Pathway Regulates Auditory Feedback of Volitional Control

**DOI:** 10.1007/s12264-025-01538-6

**Published:** 2025-12-02

**Authors:** Shengtao Luo, Yuchen Fan, Feiyang Yu, Xiaopeng Zhou, Ke Hu, Hang Yi, Hui Zhou, Tao Li, Jiang-Fan Chen, Liping Zhang

**Affiliations:** 1 The Molecular Neuropharmacology Laboratory and the Eye-Brain Research Center, State Key Laboratory of Eye Health, Eye Hospital, Wenzhou Medical University, Wenzhou, 325027 China; 2 Oujiang Laboratory (Zhejiang Laboratory for Regenerative Medicine, Vision and Brain Health), School of Ophthalmology and Optometry and Eye Hospital, Wenzhou Medical University, Wenzhou, 325027 China; 3 The Second People’s Hospital of Lishui, Lishui, 323000 China

**Keywords:** Auditory feedback, Biofeedback, Volitional control, Volitional inhibition, BCI, Secondary motor cortex (M2), External globus pallidus (GPe)

## Abstract

Effective use of brain-computer interfaces (BCIs) requires the ability to suppress a planned action (volitional inhibition) for adaptable control in real-world scenarios, but their mechanisms are unclear. Here, we used fiber photometry to monitor external globus pallidus (GPe) and subthalamic nucleus (STN) neurons' activity in mice during a volitional stop-signal task (67% GO, 33% NO-GO). GPe/STN neurons (receiving M2 projections) responded to auditory cues, feedback, and rewards in both trials. Importantly, chemogenetic activation of the M2-GPe pathway enhanced volitional inhibition by modulating auditory feedback response, yet inhibited GPe neurons’ feedback response. Furthermore, time-locked optogenetic inhibition of M2-projecting GPe neurons at auditory feedback also enhanced volitional inhibition via prolonged GO trial response times. Collectively, these findings identified the M2-GPe pathway for auditory biofeedback to improve volitional control, offering novel avenues for the advancement of neural interfaces for biofeedback and enhancement of BCI efficacy.

**Supplementary Information:**

The online version contains supplementary material available at 10.1007/s12264-025-01538-6.

## Introduction

Brain-computer interfaces (BCIs) hold great promise for assisting individuals with paralysis in regaining certain brain-controlled functions by operating neuroprosthetic devices [1–3]. To effectively utilize BCIs in real-world situations, the user must be capable of flexible control across various environments, including the ability to inhibit intended responses in accordance with environmental changes (i.e., volitional inhibition). The stop-signal task is commonly used to investigate response inhibition in motor or response control [4–7]. This paradigm requires participants to perform a 'GO' task, in which they must quickly respond to a GO stimulus. If a NO-GO stimulus follows the GO stimulus, the NO-GO stimulus instructs participants to suppress their response to the initial GO stimulus. However, beyond the GO and NO-GO stimuli, BCIs also require online feedback to turn the BCI into a bidirectional closed-loop system [8–10]. In fact, sensory stimulation and sensory feedback play important roles in BCI applications, with sensory stimulation serving as an external factor and sensory feedback indicating responses to the internal neuronal state.

The neural circuitry underlying the influence of sensory stimulation and sensory feedback on the volitional inhibition remains poorly understood. In rodents, the secondary motor cortex (M2) is analogous to the premotor cortex, supplementary motor area, or frontal eye field found in monkeys [11]. Studies have revealed the vital role of M2 in linking sensory information from the visual, auditory, and somatosensory cortices to motor functions [11–13]. Additionally, studies using optogenetics, chemogenetics, and lesion studies have demonstrated that M2 neurons process various sensory signals to regulate visual and auditory processing, guide action execution, select adaptive behaviors, and support flexible voluntary control [11, 14–16]. M2 neurons also project directly to arkypallidal and prototypic neurons in the external globus pallidus (GPe) and preferentially innervate arkypallidal neurons [17]. These prototypic and arkypallidal neurons in the GPe work together with the direct, indirect, and hyperdirect pathways in the basal ganglia to participate in sensory processing [18]. These studies suggest that GPe neurons receiving projections from M2 may play a crucial role in integrating auditory, visual, and somatosensory inputs to support volitional control.

To explore the influence of sensory input and biofeedback on the adaptability of volitional control, we designed a volitional stop-signal (GO, NO-GO) task. This task was based on a novel neuroprosthetic paradigm involving head-fixed mice, in which the mice exerted volitional control over the population calcium signals in the primary motor cortex (M1) using a fiber photometry system. In the GO trials, mice were required to respond to a 5000 Hz auditory cue within 30 seconds to intentionally adjust M1 neuronal activity above a set threshold in order to receive a reward. In the NO-GO trial, after the 5000 Hz GO sound cue, a 27,000 Hz STOP cue was presented two seconds later, requiring the mice to keep M1 neural activity below a specified threshold for 13 seconds. In this volitional stop-signal task, GO and NO-GO trials made up 67% and 33% of the total trials, respectively, and were presented randomly. We utilized anterograde transsynaptic tracing methods, along with optogenetics, chemogenetics, and combined with multi-channel and multi-color single-channel fiber photometry systems to assess the role of GPe receiving projection from M2 in regulating volitional control through the modulation of auditory feedback.

## Material and Methods

### Animals

*Thy1*-GCaMP6f transgenic mice were obtained from The Jackson Laboratory (JAX Stock No. 025393), as described previously [19]. *Thy1*-GCaMP6f transgenic mice were maintained under a 12 h/12 h light–dark photoperiod (lights on at 08.00 h). After surgery, the mice were placed on a 37 °C homeothermic pad until waking. Then *Thy1*-GCaMP6f transgenic mice were individually housed under a 12 h light–dark cycle for at least 14 days before conducting any further experiments. After the completion of the protocols, all mice were sacrificed by anesthetic overdose and cervical dislocation. All animal procedures were conducted in accordance with the National Institutes of Health Guide for the Care and Use of Laboratory Animals and were approved by the Institutional Animal Care and Use Committee of Wenzhou Medical University (xmsq2023-0254). Every effort was made to minimize animal suffering and reduce the number of animals used while maintaining scientific rigor.

### Animal Group and Number of Animals

Calcium signals were recorded from five *Thy1*-GCaMP6f mice in which the fluorescent calcium sensor jRGECO1a was expressed in the GPe and subthalamic nucleus (STN) during the GO/NO-GO task. For the chemogenetic manipulation of M2-projected GPe neurons, the experimental groups comprised six in the control group, six in the hM3D group, and six in the hM4D group. For the optogenetic manipulation of M2-projected GPe neurons, the experimental groups comprised six in the control group, seven in the eNpHR group.

### Surgery, Virus Injection, and Optic Fiber Implantation

*Thy1*-GCaMP6f transgenic mice were anesthetized with pentobarbital (60 mg/kg, i.p.) and mounted on a stereotaxic apparatus. *Thy1*-GCaMP6f transgenic mice were unilaterally injected with 300 nL of the ScAAV-hSyn-CRE-WPREs (5.40 × 10^12^ vg/mL) into the left M2 cortex (distance from bregma: anteroposterior (AP): 1.94 mm, mediolateral (ML): 0.85 mm, dorsoventral (DV): 0.85 mm). Meanwhile, *Thy1*-GCaMP6f transgenic mice were unilaterally injected with 200 nL of the rAAV-EF1α-DIO-jRGECO1a-WPREs into the left GPe nucleus (AP: − 0.34 mm, ML:1.90 mm, DV: − 3.50 mm) and left STN nucleus (AP: − 2.06 mm, ML: − 1.55 mm, DV: − 4.30 mm), respectively, for calcium imaging using a Nanojet II injector. For chemogenetic manipulation, *Thy1*-GCaMP6f transgenic mice were unilaterally injected with 200 nL of the rAAV-EF1α-DIO-hM3D(Gq)-mCherry-WPREs (5.04 × 10^12^ vg/mL), rAAV-EF1α-DIO-hM4D(Gi)-mCherry-WPREs (5.34 × 10^12^ vg/mL) and rAAV-EF1α-DIO-mCherry-WPREs-hGH, respectively into the left GPe nucleus (AP: − 0.34 mm, ML: 1.90 mm, DV: − 3.50 mm) using a Nanojet II injector (Drummond Scientific, Broomall, PA, USA) at a rate of 60 nL/min. For the optogenetic manipulation, *Thy1*-GCaMP6f transgenic mice were unilaterally injected with 200 nL of the rAAV-EF1α-DIO-eNpHR3.0--mCherry-WPRE-hGH (5.14 × 10^12^ vg/mL and rAAV-EF1α-DIO-mCherry-WPREs-hGH (5.40 × 10^12^ vg/mL), respectively, into the left GPe nucleus (AP: − 0.34 mm, ML: 1.90 mm, DV: − 3.50 mm) using a Nanojet II injector (Drummond Scientific, Broomall, PA, USA) at a rate of 60 nL/min. *Thy1*-GCaMP6f transgenic mice were then implanted with an optical fibre (230 μm O.D., 0.37 Numerical Aperture (NA); Shanghai Fiblaser Technology Co., Ltd (Shanghai, China)) within a ceramic ferrule in the M1, GPe, and STN at the same sites. The ceramic ferrule was supported with a skull-penetrating screw and dental cement.

### Calcium Fluorescence Signal Analysis

Photometry data were exported to MATLAB Mat files for further analysis using the MATLAB platform (MathWorks, Natick, MA, USA) with custom-written programs [20–22]. After smoothing the data with a moving average filter (20 ms span with a 10 ms moving step), the event-related calcium fluorescence signals relating to the reward (with reward delivery set as time ‘0’) were analyzed. We derived the calcium signal change values (△*F*) by calculating △*F/F* =(*F − F*0)/*F*0, where *F*0 was the baseline of the population calcium signal at the time (within 10 ms) of the auditory cue occurrence. No recording data were excluded from the analysis.

### Data Analysis

Statistical analyses were performed using GraphPad Prism 5.01 (GraphPad Software Inc., San Diego, CA, USA). Data are expressed as means ± SEM. An unpaired two-tailed Student’s *t*-test and Mann–Whitney U test were used to compare two-group data, as appropriate. A *P*-value of <0.05 was considered statistically significant: ∗*P* < 0.05, ∗∗*P* < 0.01, ∗∗∗*P* < 0.001. In the chemogenetic manipulation experiments, the results were averaged across sessions for each mouse, resulting in *n =* 6 independent data points for subsequent statistical analysis.

### Fluorescence

A sample was performed on free-floating sections (30 μm). The sections were directly imaged using confocal laser scanning microscopy (LSM710; Carl Zeiss, Oberkochen, Germany).

### The Volitional Stop-Signal Task

**Fig. 1:**
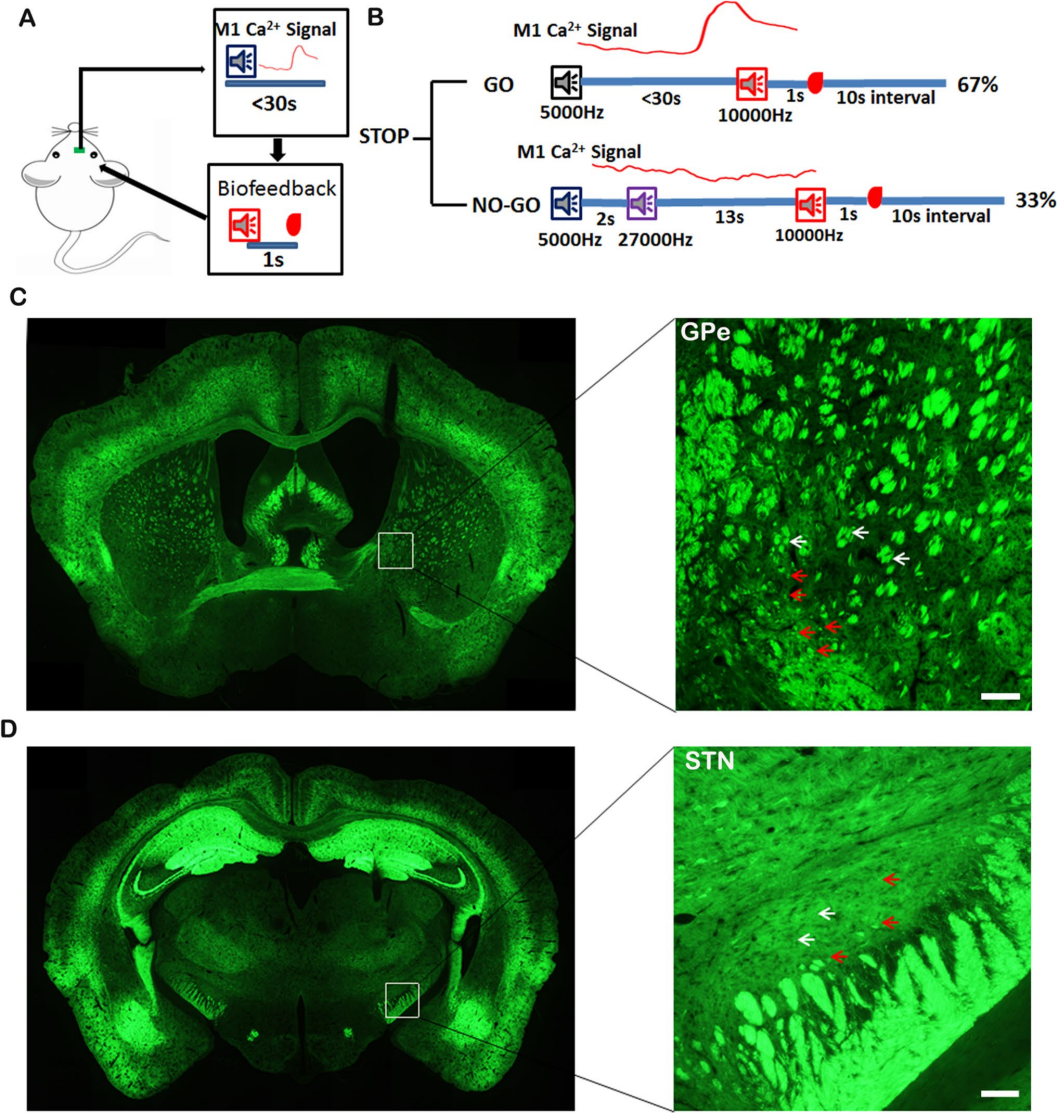
Development of the Volitional Stop-signal Task. **A** Closed-loop BCI control system: the population calcium signal (ΔF) exceeding the defined threshold value to trigger auditory feedback and deliver a reward after 1 s. **B** The volitional stop-signal task. GO and NO-GO tasks accounted for 67% and 33%, respectively. In the NO-GO task, following a GO cue, the mice were instructed to inhibit the calcium signal of M1 below a defined threshold value for a duration of 13 s after the NO-GO cue was presented, which occurred 2 s post-GO signal. In the GO task, the mice were instructed to activate the calcium signal of M1 exceeding the defined threshold value within 30 s after the GO cue was presented. **C** *Thy1*-GCaMP6f transgenic mice exhibited GCaMP6f expression in their brains. The right panel was enlarged to show the white arrow in the left mouse brain section for the GPe. The red arrows indicate neurons in GPe expressing GCaMP6f, and the white arrows indicate nerve fibers in the GPe. **D** *Thy1*-GCaMP6f transgenic mice expressed GCaMP6f in their brains. The right panel was enlarged to show the white arrow in the left mouse brain section for the STN. The red arrows indicate neurons in the STN expressing GCaMP6f, and the white arrows indicate nerve fibers in the STN. Scale bar, 100 μm.

The volitional control task has been thoroughly described in previous research [20]. *Thy1*-GCaMP6f transgenic mice first underwent training to complete 50 correct GO trials daily for 10 days, achieving a final accuracy rate of 85%–100%. They then underwent a random reward test to confirm goal-oriented volitional control. Next, these mice were trained to complete 50 correct NO-GO trials daily over another 10 days, with final accuracy also reaching 85%–100%. Once proficiency in both GO and NO-GO tasks was achieved—defined by a correct response rate of 85%–100% over three consecutive days—the *Thy1*-GCaMP6f transgenic mice were subjected to the volitional stop-signal task (Fig. 1B). This task comprises a total of 50 successful trials, with 67% being GO trials and 33% being NO-GO trials. Success rates are calculated as the proportion of successful GO trials relative to the total number of GO trials, and similarly for NO-GO trials. The response time for both GO and NO-GO trials is defined as the interval from trial onset to reaching the preset threshold.

## Result

### Volitional Stop-signal Paradigm

In our previous study, we developed an operant, volitionally controlled neural task integrated with a closed-loop feedback system. This system enabled volitional conditioning of population neurons in the M1 cortex *via* real-time monitoring of calcium fluorescence signals using a fiber photometry system [20, 22]. For the current study, we improved the previous volitional control task by utilizing different auditory frequencies as cues and biofeedback independently (Fig. 1A). In the GO task, mice were exposed to a 5,000 Hz auditory stimulus to conditionally influence M1 neuronal activity to cross a specified threshold within 30 seconds. Subsequently, 10,000 Hz auditory feedback was provided upon the calcium signal crossing the preset threshold, followed by a 1 s delay before receiving a sucrose reward, and then a 10 s interval before the next trial began (Fig. 1B, upper). In the NO-GO task, mice were exposed to a 5000 Hz auditory stimulus and a 2 s wait for the second auditory stimulus (27,000 Hz). Then, mice were required to maintain M1 neuronal activity below threshold for 13 s. Successful maintenance triggered a 10,000 Hz auditory feedback tone (500 ms duration), followed after 1 s by sucrose reward delivery. A 10-s inter-trial interval preceded the next trial (Fig. 1B, below). The volitional stop-signal paradigm comprised 67% GO tasks and 33% NO-GO tasks, with each trial type occurring randomly (Fig. 1B). For the present investigation, we employed a fiber photometry system for real-time monitoring of calcium fluorescence signals in the M1 cortex of *Thy1*-GCaMP6f transgenic mice, facilitating the volitional conditioning of neuronal populations. *Thy1*-GCaMP6f transgenic mice were engineered to express GCaMP6f in various brain regions, including the cortex, hippocampus, amygdala, thalamus, and hypothalamus, among others [19] (Fig. 1C, D). Our investigation revealed that many GPe and STN neurons (Fig. 1C, D, right panel: red arrow) exhibited GCaMP6f expression, whereas a large number of nerve fibers in these regions also exhibited GCaMP6f expression (Fig. 1C, D, right panel: white arrow).

### M2-innervated GPe and STN Neurons Respond to Auditory Cue, Reward, and Auditory Feedback in the Volitional Control

**Fig. 2:**
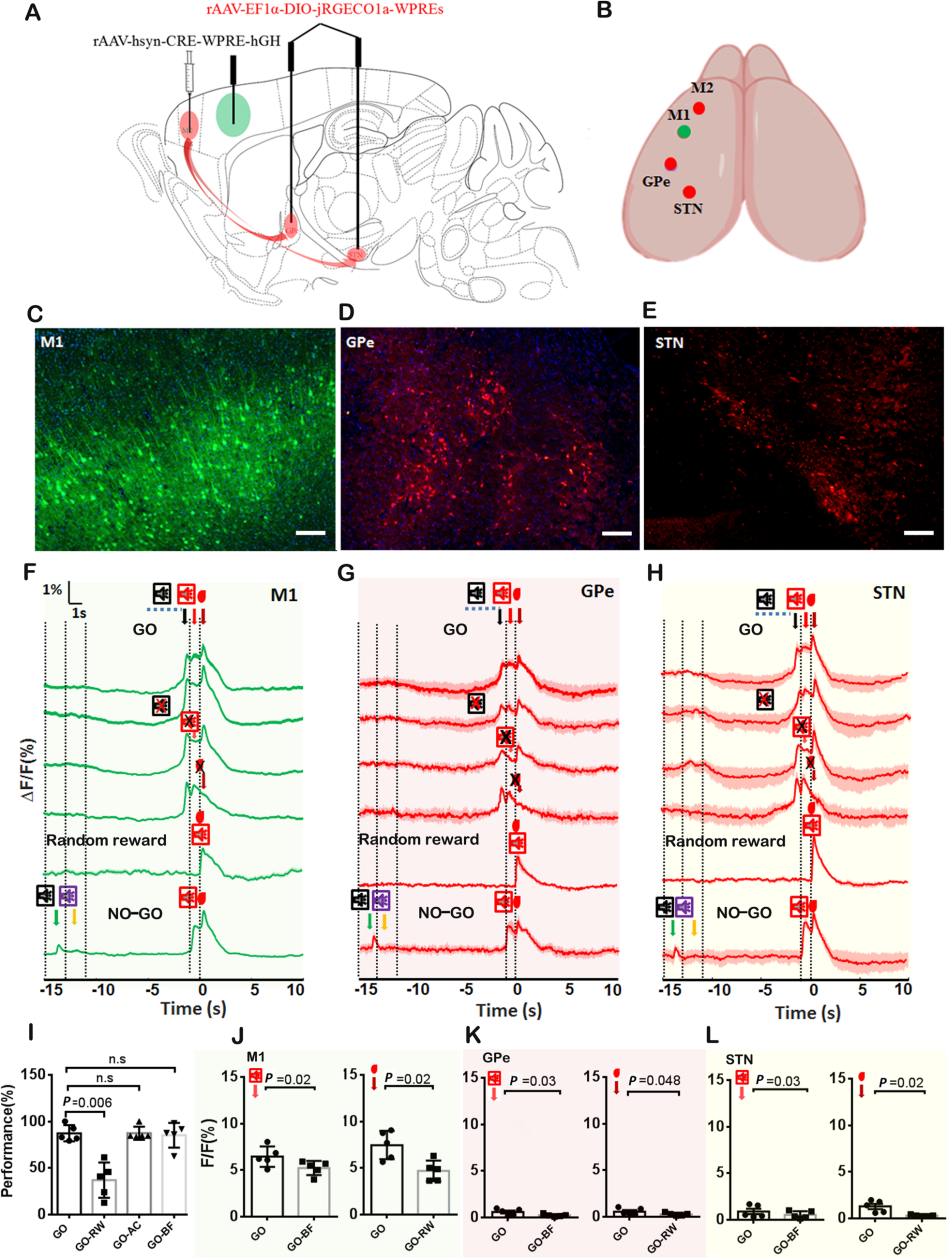
The Change of Calcium Signal for M2 Projected GPe /STN Neurons in GO, NO-GO Task. **A** Schematic of the injection for anterograde transsynaptic tracing. ScAAV-hSyn-CRE-WPREs were injected into the M2, and rAAV-EF1α-DIO-jRGECO1a-WPREs were injected into the GPe and STN. **B** M1, M2, GPe, and STN were located on the same hemisphere of the mouse brain. **C** *Thy1*-GCaMP6f transgenic mice exhibit expression of GCaMP6f in M1. **D** The expression of jRGECO1a in the GPe by anterograde transsynaptic tracing. **E** The expression of jRGECO1a in STN by anterograde transsynaptic tracing. **F** Analysis of a representative population calcium signal before (15 s) and after (10 s) the reward delivery for M1 in GO, GO withdraw GO cue, GO withdraw auditory feedback, GO withdraw reward, random reward, and NO-GO task. Different calcium signals are indicated by the different color arrows. Black arrow: the volitional signal; Red arrow: the auditory feedback signal; Dark red arrow: reward signal; Green arrow: the GO auditory cue signal; Yellow arrow: the Stop auditory cue signal. **G** Same as F, but for GPe. **H** Same as F, but for STN. **I** The performance ON the GO task, GO withdraw, GO cue, GO withdraw auditory feedback, GO withdraw reward (*n* = 5). GO-BF: GO withdraw auditory feedback; GO: GO task; GO-RW: GO withdraw reward; GO-AC: GO withdraw auditory cue. **J** The analysis of the peak of the calcium signal for auditory feedback and reward in M1 between the GO task and GO withdraw auditory feedback( left) or GO withdraw reward. **K** Same as J, but for GPe. L) Same as J, but for STN. Scale bar, 100 μm. Sample size: *n* = 6.

To investigate the neural mechanisms regulating responses to auditory cues and feedback, we used anterograde transsynaptic tracing to label GPe and STN neurons that receive M2 projections, and expressed the fluorescent calcium sensor jRGECO1a in these labeled neurons (Fig. 2A, B). As demonstrated in Fig. 2C–E, GCaMP6f expression in the M1 region of *Thy1* transgenic mice (Fig. 2C) and jRGECO1a expression in neurons located in the GPe (Fig. 2D) and the STN (Fig. 2E) confirmed that GPe and STN neurons receive input from M2 neurons. We trained the mice to modulate M1 neurons to execute GO and NO-GO tasks (Fig. 1B) while simultaneously monitoring calcium signal changes in the GPe and STN in real-time using multi-color single-channel fiber photometry systems. The calcium signals were aligned to the time of reward delivery(“0”) and represented the calcium signal peak before (15 s) and after (10 s) reward delivery. During the GO task, calcium signals corresponding to volitional control, auditory feedback, and reward were detected in the M1, GPe, and STN (Fig. 2F–H). To validate these signals, we systematically removed the auditory cue, auditory feedback, or reward from the GO task, one at a time. As shown in Fig. 2F–H, the removal of auditory feedback or reward during the GO task results in the disappearance of the associated signal in the M1, GPe, and STN (Fig. 2F–H). The results also revealed that only withdrawal of the reward was sufficient to alter performance in the GO task (Fig. 2I, *P* = 0.006). We conducted a comparative analysis of the alterations in calcium signal following the removal of auditory cues, reward, and auditory feedback individually. Our findings indicated that the calcium signal exhibited a significant reduction upon the removal of each of these stimuli in M1 (Fig. 2J; left: *t*-test *P *= 0.02; Right: *t*-test *P *= 0.02), GPe (Fig. 2K, left: *t*-test *P *= 0.03; Right: *t*-test *P *= 0.048), and STN (Fig. 2L, left: *t*-test *P *= 0.03; Right: *t*-test *P*=0.02). The volitional signal is characterized as the calcium signal that occurs prior to attaining the set threshold. However, given that the interval between the volitional signal and the auditory cue is variable during the GO task, the auditory cue signal is not detected in the M1, GPe, and STN. In the NO-GO task, we noted the presence of the GO auditory cue, auditory feedback, and reward signal; however, the stop auditory cue signal was not detected in the M1, GPe, and STN regions (Fig. 2F–H). In the random reward test, we also observed a combined calcium signal of the auditory cue and reward. Taken together, we inferred that M1 neurons, GPe, and STN neurons innervated by M2 responded to auditory cues, auditory feedback, and reward during both GO and NO-GO tasks.

### Chemogenetic Activation of M2-GPe Pathway Promotes Volitional Inhibition

**Fig. 3:**
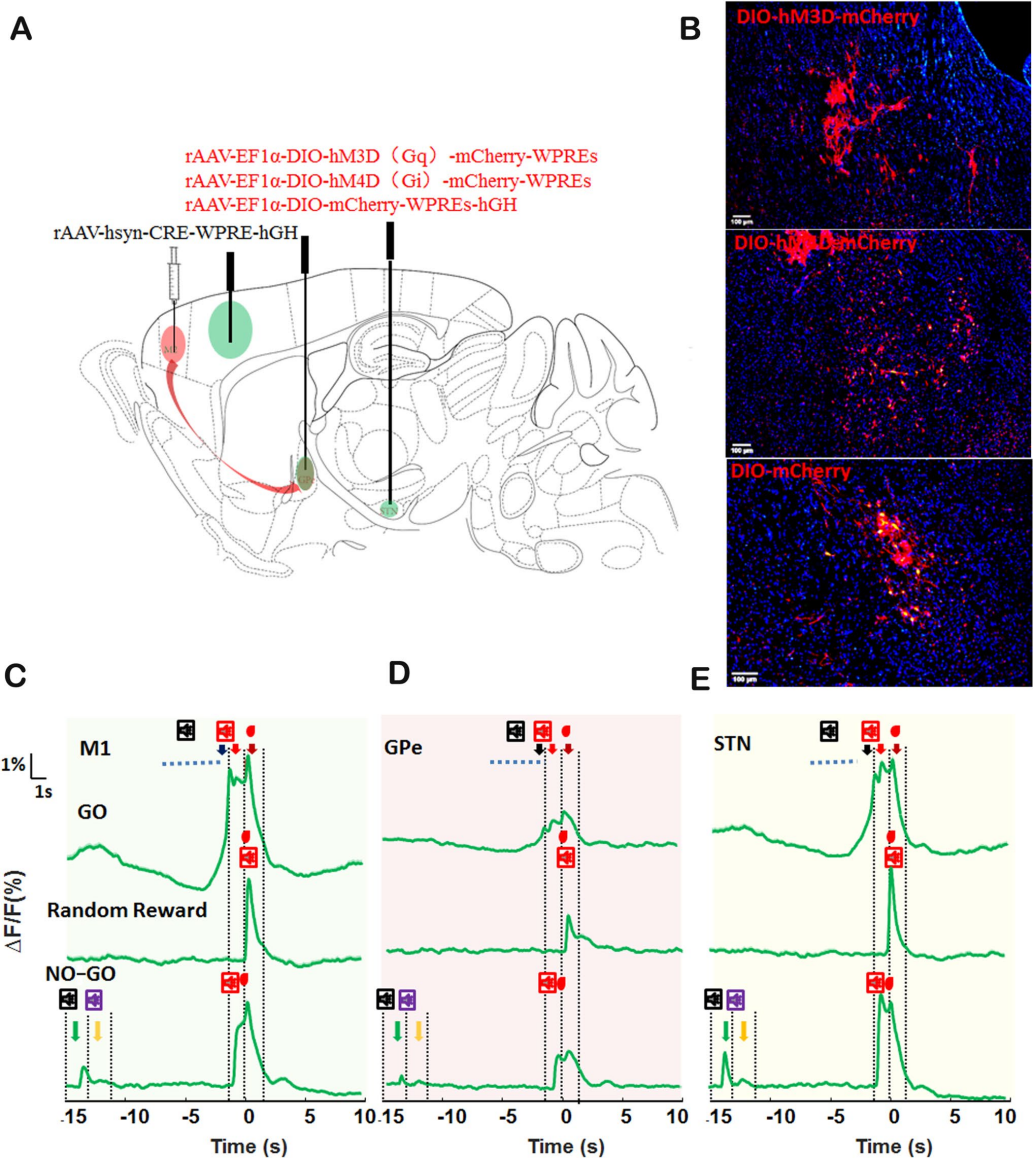
Schematic of the Injection for Anterograde Transsynaptic Tracing and Calcium Imaging. **A** ScAAV-hSyn-CRE-WPREs was injected into the M2, and rAAV-EF1α-DIO-hM3D(Gq)-mCherry-WPREs/ rAAV-EF1α-DIO-hM4D(Gi)-mCherry-WPREs/ rAAV-EF1α-DIO-mCherry-WPREs-hGH were injected into the GPe. Fiber optic probes were implanted in the M1, GPe, and STN to facilitate the monitoring of calcium signaling in *Thy1*-GCaMP6f transgenic mice. **B** The hM3D(Gq), hM4D(Gi), and mCherry expression in the GPe using *Thy1*-GCaMP6f transgenic mice. **C** Analysis of a representative population calcium signal before (15 s) and after (10 s) the reward delivery for M1 in the GO, random reward, and NO-GO task using *Thy1*-GCaMP6f transgenic mice. Different calcium signals are indicated by the different color arrows. Black arrow: the volitional signal; Red arrow: the auditory feedback signal; Dark red arrow: reward signal; Green arrow: the GO auditory cue signal; Yellow arrow: the Stop auditory cue signal. **D** Same as C, but for the GPe. **E** Same as C, but for STN. Scale bar, 100 μm.

**Fig. 4:**
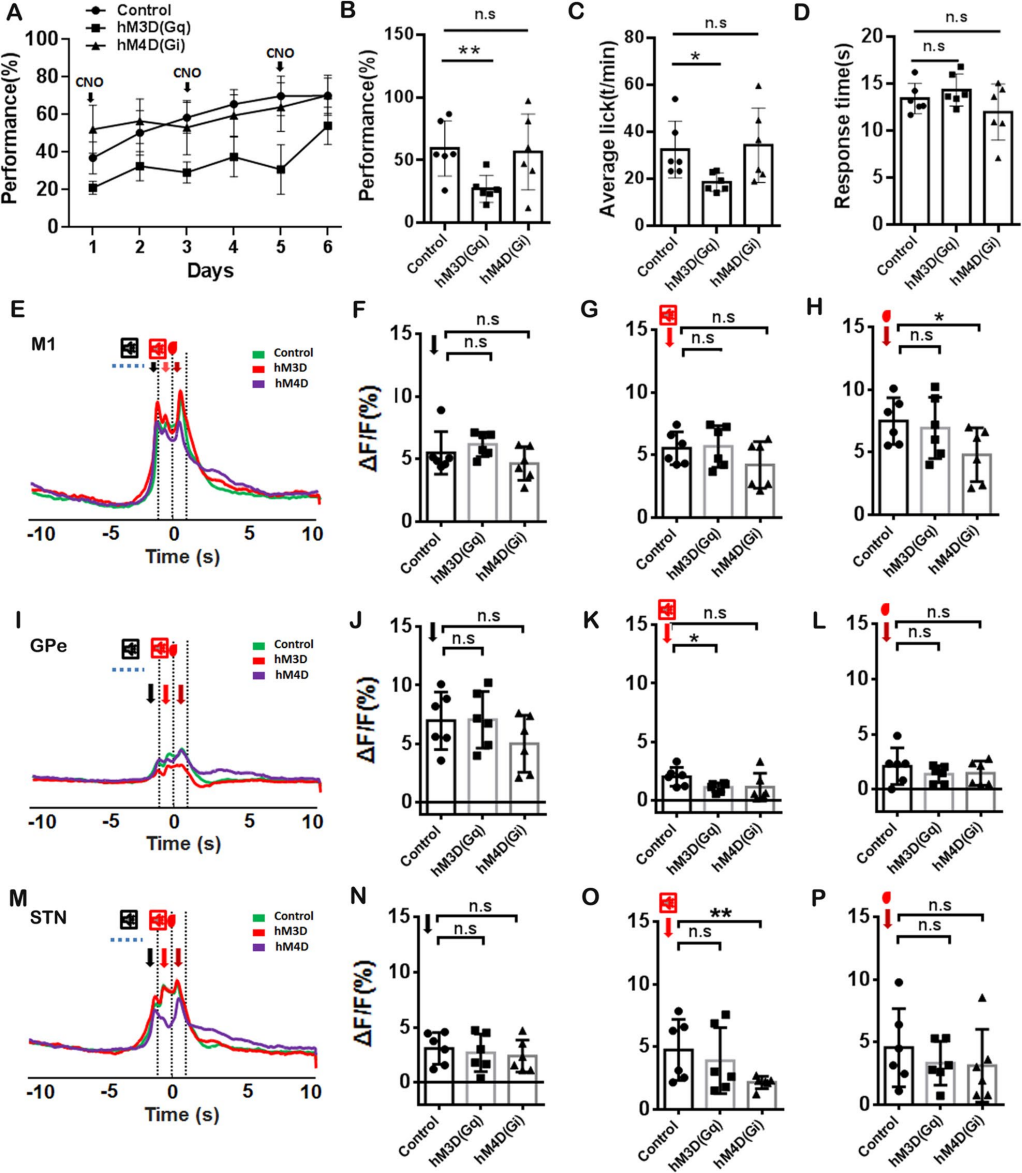
Chemogenetic Manipulation of M2-innervated GPe Neurons in the Volitional Stop Signal Task. **A** The performance of GO tasks with or without manipulation of GPe neurons for 6 days in the volitional stop signal task. **B** Analysis of the performance on the GO task for a total of 3 days among the control group, hM4D(Gi), and hM3D(Gq) groups. **C** Analysis of the total licks of the GO task for 3 days among the control group, hM4D(Gi), and hM3D(Gq) groups. **D** Analysis of response time of the GO task for 3 days among the control group, hM4D(Gi), and hM3D(Gq)group. **E** A representative population calcium signal before (15 s) and after (10 s) the reward delivery for M1 in control, hM4D(Gi), and hM3D(Gq) groups using *Thy1*-GCaMP6f transgenic mice. **F** Analysis of the peak of the calcium signal for the volitional signal among control, hM4D(Gi), and hM3D(Gq) groups. **G** Analysis of the peak of the calcium signal for auditory feedback among control, hM4D(Gi), and hM3D(Gq)groups. **H** Analysis of the peak of the calcium signal for the reward signal among control, hM4D(Gi), and hM3D(Gq)groups. **H–K** Same as **D–G**, but for the GPe. **L–O** Same as **D–G**, but for the STN. The control group: *n* = 6; hM4D(Gi) group:* n* = 6; hM3D(Gq)group:* n* = 6.

To illuminate the volitional inhibition function of GPe neurons innervated by M2, we employed anterograde transsynaptic tracing techniques to label the input from M2 to GPe using hM3D(Gq) or hM4D(Gi), respectively(Fig. 3A, B). Since GPe neurons receiving projection from M2 were already labeled with hM3D(Gq) or hM4D(Gi) and could not be simultaneously labeled with the fluorescent calcium sensor jRGECO1a (red), we directly recorded the GPe and STN neuronal calcium signals of *Thy1*-GCaMP6f transgenic mice using multi-channel fiber photometry systems. Nevertheless, we also identified the volitional, auditory feedback, and reward signals within the M1, GPe, and STN, which exhibited a similar pattern to that observed in M1, GPe, and STN neurons receiving projection from M2 (Fig. 3C–E). We also observed a calcium signal for the stop auditory cue (27,000Hz at GPe and STN) in the NO-GO task (Fig. 3C–E). Consequently, we believe that some neurons identified within the GPe and STN of *Thy1*-GCaMP6f transgenic mice received M2 projections. We, subsequently, carried out a six-day experimental procedure during which Clozapine N-oxide (CNO) was administered on alternate days, culminating in a total of three administration days (Fig. 4A). We calculated the results of three days of CNO treatment and found that the activation of GPe neurons innervated by M2 decreased the GO task success rate (Fig. 4B; *P <*0.01). However, the inhibition of these neurons did not affect the GO task (Fig. 4B, *P >*0.05). The pharmacological activation of GPe neurons receiving projection from M2 also inhibited licking behavior in the GO task (Fig. 4C, *P <*0.05), but pharmacological inhibition of these neurons had no such influence on the licking behavior (Fig. 4C, *P >*0.05). Nonetheless, the pharmacological activation and inhibition of these neurons did not influence response time in the GO task, indicating that the inhibition of GO success by activating GPe neurons innervated by M2 was not achieved by altering response time during the volitional stop-signal paradigm (Fig. 4D, *P >*0.05).

We also analyzed the calcium signal for the volitional, auditory feedback, and reward in the M1, GPe, and STN (Fig. 4E–P). To eliminate the influence of different transgenic mouse models on GCaMP6f expression levels, we analyzed the calcium signal in response to auditory cues, volitional, auditory feedback, and reward among control, HM3D (Gq), and HM4D (Gi) groups during the GO and NO-GO tasks in the absence of CNO treatment (supplemental Fig. S1–S2). In addition to the significant differences in auditory feedback observed between the hM4D group and the control groups within the STN in the GO task (supplemental Fig. S1K, *P <*0.05) and the NO-GO task (supplemental Fig. S2N, *P <*0.05), no substantial variations were identified in auditory cues, volitional signals, auditory feedback, or reward signals in the M1 and GPe. The inhibition of GPe neurons innervated by M2 significantly inhibited the calcium signal for reward signal in M1 (Fig. 4H, *P <*0.05), for auditory feedback in STN (Fig. 4O, *P <*0.01). However, the activation of GPe neurons innervated by M2 only significantly inhibited the calcium signal for the auditory feedback in the GPe during the GO task (Fig. 4F–H, J, L, N–P, *P >*0.05; Fig. 4K; *P <*0.05).

**Fig. 5:**
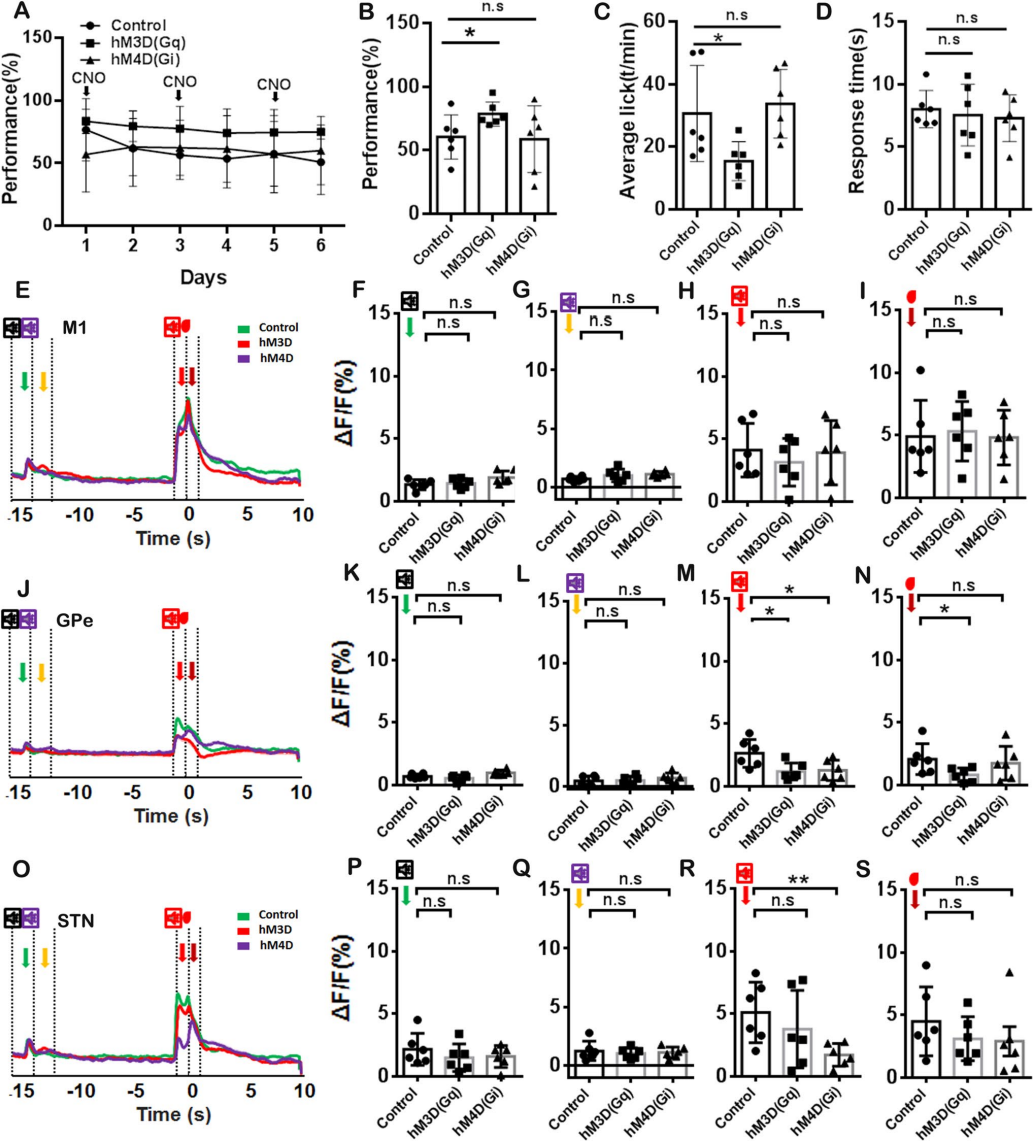
Analysis of the Performance and Calcium Signal for the NO-GO Task in the Volitional Stop Signal Task. **A** The performance on NO-GO tasks with or without manipulation of GPe neurons for 6 days in the volitional stop signal task. **B** Analysis of the performance on the NO-GO task for a total of 3 days among the control, hM4D(Gi), and hM3D(Gq) groups. **C** Analysis of the total licks of the GO task for 3 days among the control, hM4D(Gi), and hM3D(Gq) groups. D) Analysis of response time of error NO-GO task for 3 days among the control, hM4D(Gi), and hM3D(Gq) groups. **D** Analysis of a representative population calcium signal before (15 s) and after (10 s) the reward delivery for M1 in control, hM4D(Gi), and hM3D(Gq) groups using *Thy1*-GCaMP6f transgenic mice. **E** Analysis of the peak of the calcium signal for the volitional signal among control, hM4D(Gi), and hM3D(Gq) groups. **F** Analysis of the peak of the calcium signal for auditory feedback among control, hM4D(Gi), and hM3D(Gq) groups. **G** Analysis of the peak of the calcium signal for the reward signal among the control, hM4D(Gi), and hM3D(Gq) groups. **H–K** Same as **D**–**G**, but for the GPe. **L–O** Same as **D–G**, but for the STN. The control group: *n =* 6; hM4D(Gi) group: *n =* 6; hM3D(Gq) group: *n =* 6.

Next, we analyzed the performance, licking behavior, response time, and calcium signals for the NO-GO task in the volitional stop-signal paradigm (Fig. 5A–S). The results indicated the activation of these neurons enhanced performance on the NO-GO task (Fig. 5A and B, *P<*0.05) and decreased licking behavior (Fig. 5C, *P* *<*0.05), but had no influence on the error-trial response time (Fig. 5D; *P >*0.05). The inhibition of GPe neurons innervated by M2 did not affect performance (Fig. 5B, *P* *>*0.05), licking behavior (Fig. 5C, *P >*0.05), and error-trial response time (Fig. 5D, *P >*0.05). The analysis of calcium signal indicated that the activation of these neurons inhibited the calcium signal for auditory feedback (Fig. 5M, *P <*0.05) and reward in the GPe (Fig. 5N, *P <*0.05). The inhibition of these neurons also decreased the calcium signal for auditory feedback in the GPe (Fig. 5M, *P <*0.05) and for auditory feedback (Fig. 5R, *P <*0.01) in the STN. Taking the above results together, we suggest that GPe neurons receiving projection from M2 play a crucial role in modulating auditory feedback during the volitional stop-signal task to affect volitional inhibition.

### GPe Neurons Receiving Projection From M2 Regulate Auditory Feedback to Enhance Volitional Inhibition

**Fig. 6:**
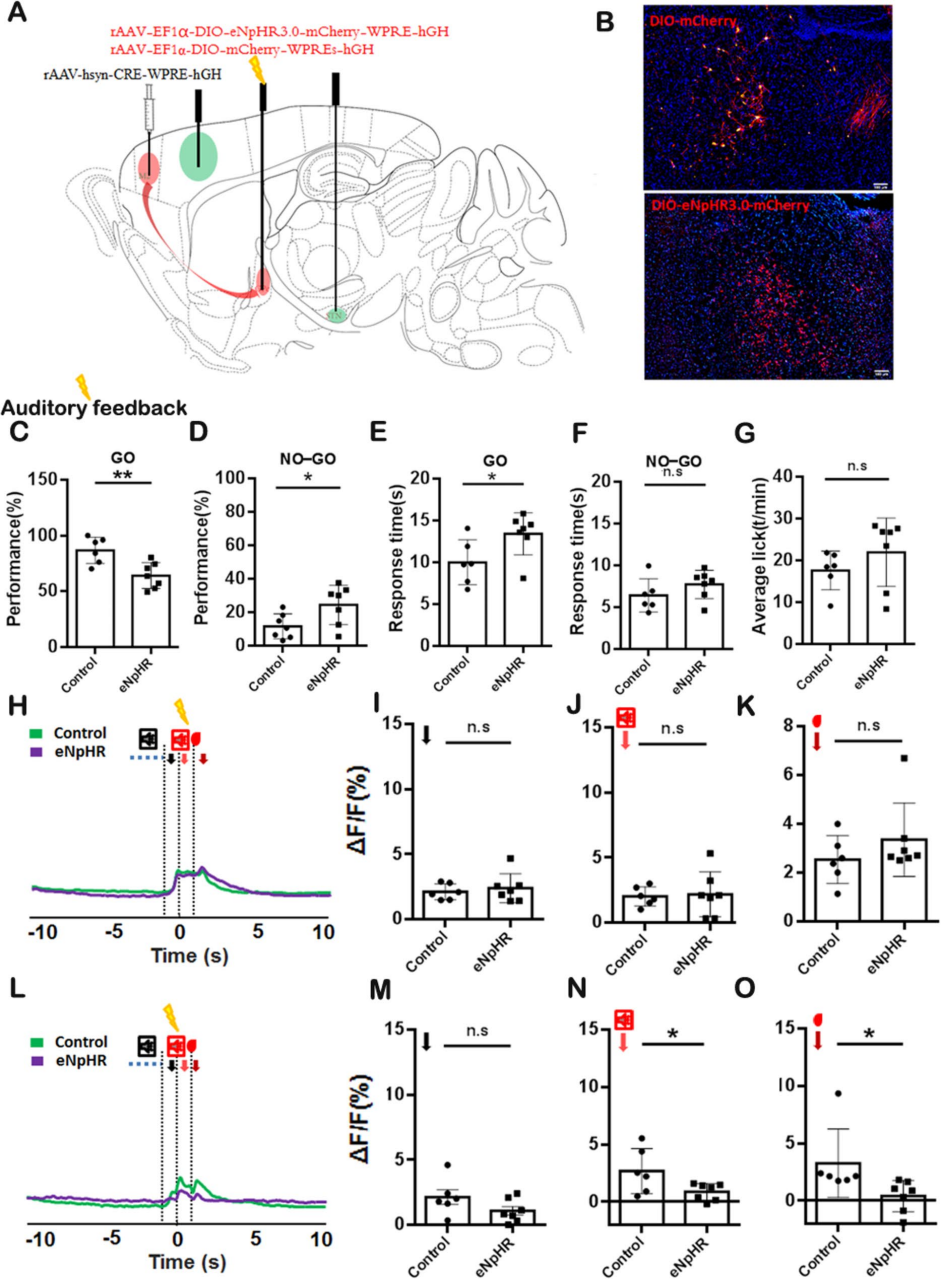
Optogenetic Inhibition of M2-innervated GPe Neurons in the Volitional Stop Signal Task during Auditory Feedback. **A** Schematic of the injection for anterograde transsynaptic tracing. ScAAV-hSyn-CRE-WPREs were injected into the M2, and rAAV-EF1α-DIO-eNpHR3.0--mCherry-WPRE-hGH or rAAV-EF1α-DIO-mCherry-WPREs-hGH were injected into the GPe. Fiber optic probes were implanted in the M1 and STN to facilitate the monitoring of calcium signaling and implanted in GPe for optogenetic manipulation in *Thy1*-GCaMP6f transgenic mice. **B** Representative image of eNpHR3.0 and mCherry expression in GPe. **C** The GO performance analysis between the control and eNpHR3.0 groups. **D** Analysis of the performance on the NO-GO task between the control and eNpHR3.0 group in the volitional stop signal task. **E** The response time on the GO task between the control and eNpHR 3.0 groups in the volitional stop signal task. **F** The response time on the NO-GO task between the control and eNpHR 3.0 group in the volitional stop signal task. **G** The analysis of licking behavior between the control and eNpHR3.0 group in the volitional stop signal task. **H** The representation of calcium signal before (10 s) and after (10 s) the reward delivery for M1 using *Thy1*-GCaMP6f transgenic mice. **I** Analysis of the peak calcium signal for the volitional signal between control and eNpHR groups. **J** Analysis of the peak calcium signal for auditory feedback between control and eNpHR groups. **K** Analysis of the peak of the calcium signal for reward between control and eNpHR groups. **L–O** Same as **H–K**, but for STN. The control group: *n =* 6; eNpHR 3.0 group: *n =* 7.

To further verify the function of GPe neurons innervated by M2, we labeled GPe neurons receiving projection from M2 by employing anterograde transsynaptic tracing techniques using eNpHR (Fig. 6A-B). We employed optogenetic techniques for time-licked inhibiting labelled GPe neurons for a duration of 1 s in conjunction with auditory feedback during the volitional stop-signal paradigm. The results indicated that the transient optogenetic inhibition of GPe neurons receiving projection from M2 resulted in a decreased success rate in the GO task (Fig. 6C, *P <*0.01), as well as an increased response time required to complete the task (Fig. 6E, *P <*0.05). Prolonged response time is a well-validated behavioral marker of response inhibition, reflecting the active suppression of prepotent motor plans—a core feature of volitional stop processes [23]. However, the transient optogenetic inhibition of GPe neurons innervated by M2 increased the NO-GO success rate (Fig. 6D, *P <*0.05), but had no influence on the response time for the error-trial (Fig. 6F, *P >*0.05). Simultaneously, the transient optogenetic inhibition of GPe neurons receiving projection from M2 did not appear to affect licking behavior (Fig. 6G, *P >*0.05). Analysis calcium signal indicated that the calcium signals for auditory feedback and reward exhibited a significant difference in the STN (Fig. 6N, *P <*0.05; Fig. 6O, *P <*0.05), but have no influence on the volitional signal, auditory feedback and reward in M1 (Fig. 6H–K, *P >*0.05) and on the volitional signal in STN (Fig. 6M, *P >*0.05). In summary, the modulation of volitional inhibition via the M2-GPe pathway is accomplished through the prolongation of response times in GO tasks.

**Fig. 7:**
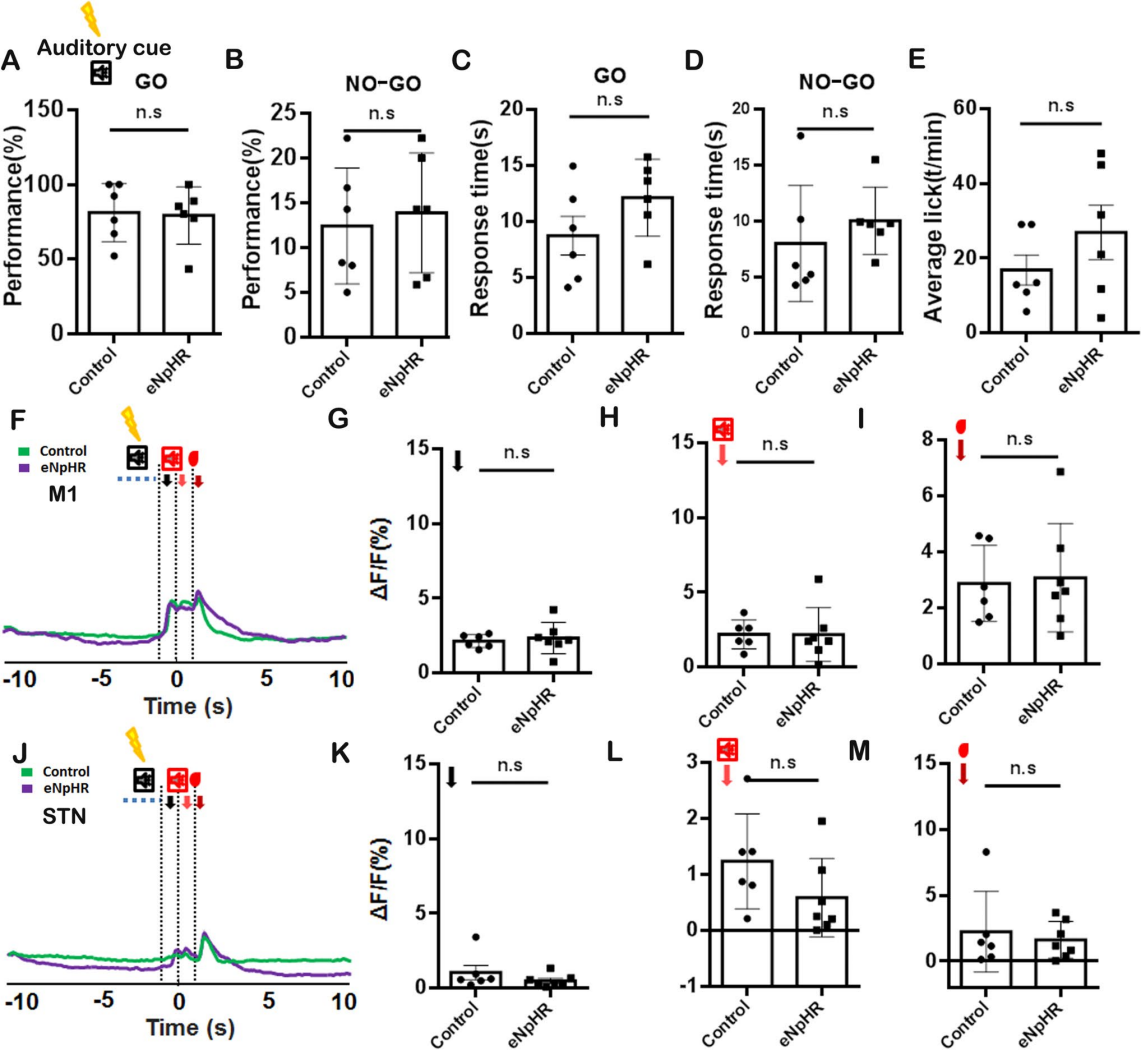
Optogenetic Inhibition of M2-innervated GPe Neurons during Auditory Cue Presentation. **A** The analysis of GO task performance between the control and eNpHR3.0 group. **B** The analysis of NO-GO task performance between the control and eNpHR3.0 groups in the volitional stop signal task. **C** The response time analysis for the GO task between the control and eNpHR 3.0 group in the volitional stop signal task. **D** The response time analysis for the NO-GO task between the control and eNpHR 3.0 groups in the volitional stop signal task. **E** The analysis of licking behavior between the control and eNpHR3.0 group in the volitional stop signal task. **F** The representation of calcium signal before (10 s) and after (10 s) the reward delivery for M1 using *Thy1*-GCaMP6f transgenic mice. **G** Analysis of the peak calcium signal for the volitional signal between control and eNpHR groups. **H** Analysis of the peak calcium signal for auditory feedback between control and eNpHR groups. **I** Analysis of the peak calcium signal for reward between control and eNpHR groups. **J–M** Same as F-I, but for the STN. The control group: *n =* 6; eNpHR 3.0 group: *n =* 7.

**Fig. 8:**
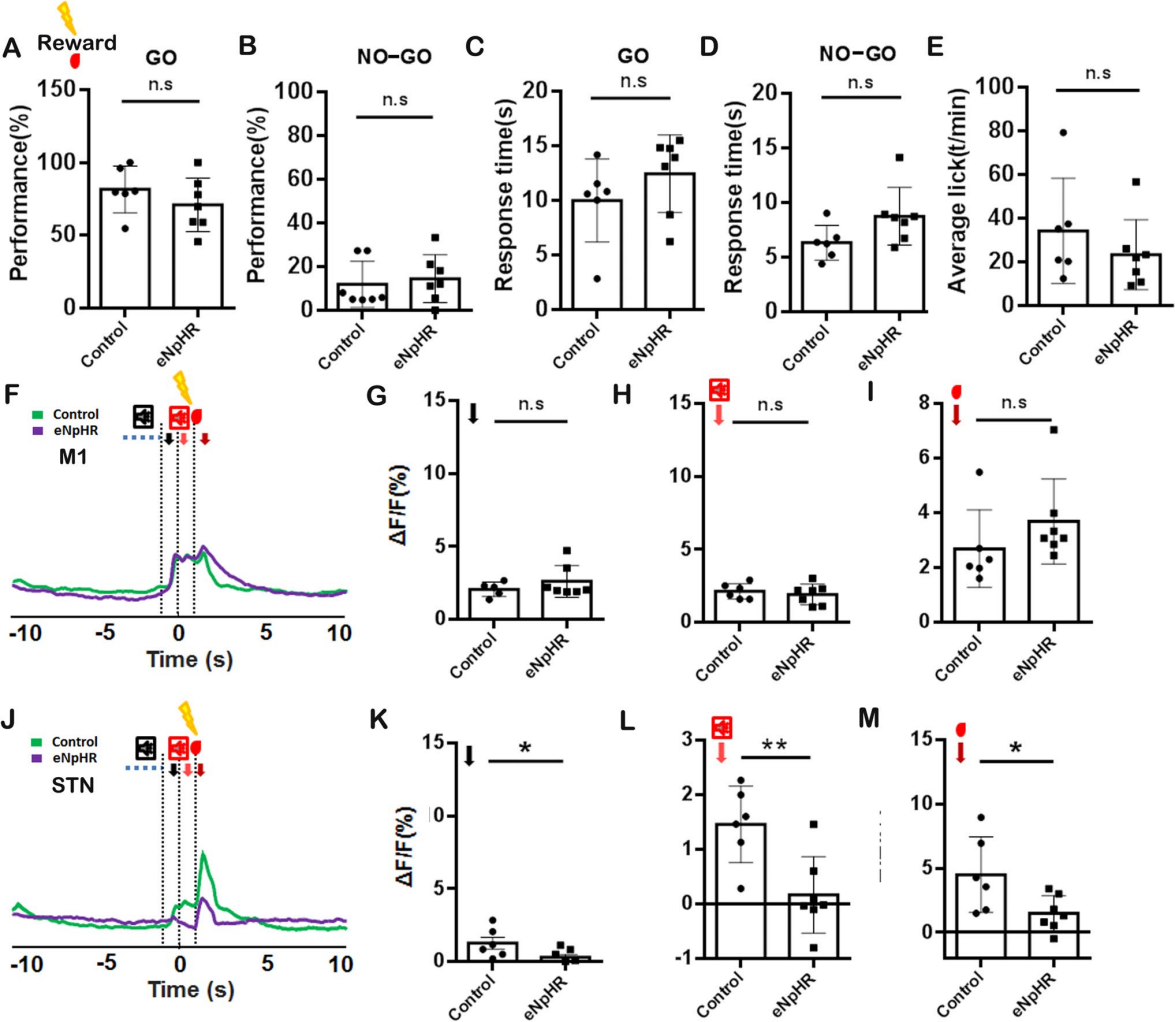
Optogenetic inhibition of M2-innervated GPe neurons during reward presentation. **A** The GO task performance analysis between the control and eNpHR3.0 groups. **B** The NO-GO task performance analysis between the control and eNpHR3.0 groups in the volitional stop signal task. **C** Analysis of the GO task response time between the control and eNpHR 3.0 groups in the volitional stop signal task. **D** Analysis of the go task response time between the control and eNpHR 3.0 groups in the volitional stop signal task. **E** Analysis of licking behavior between the control and eNpHR3.0 groups in the volitional stop signal task. **F** The representation of calcium signal before (10 s) and after (10 s) the reward delivery for M1 using *Thy1*-GCaMP6f transgenic mice. **G** Analysis of the peak of the calcium signal for the volitional signal between control and eNpHR groups. **H** Analysis of the peak of the calcium signal for auditory feedback between control and eNpHR groups. **I** Analysis of the peak of the calcium signal for reward between control and eNpHR groups. J–M) Same as F–I, but for STN. The control group: *n =* 6; eNpHR 3.0 group: *n =* 7.

However, we also employed transient optogenetic inhibition of GPe neurons innervated by M2 during auditory cue (Fig. 7A–M) and reward presentation (Fig. 8A–M) for a duration of 1 s in the volitional stop-signal test. The optogenetic inhibition of GPe neurons receiving projection from M2 neurons in response to GO auditory cue or rewards during the volitional stop-signal paradigm did not affect performance on GO (Fig. 7A, *P* >0.05; Fig. 8A, *P* >0.05) and NO-GO (Fig. 7B, *P* >0.05; Fig. 8B, *P* >0.05) tasks, response time (Fig. 7C, D, *P* >0.05; Fig. 8C, D, *P* >0.05) and licking behavior (Fig. 7E, *P* >0.05; Fig. 8E, *P* >0.05).

We analyzed the calcium signaling in response to volitional signals, auditory feedback, and reward signals during the GO tasks of control and eNpHR groups in the absence of optogenetic inhibition. No significant differences were observed between the control and eNpHR groups in terms of the calcium signaling the M1 and STN regions (Supplemental Fig. 3). Transient optogenetic inhibition of M2-innervated GPe neurons during auditory cue presentation did not significantly alter calcium signals associated with volitional action, auditory feedback, or reward delivery in either M1 or STN (Fig. 7F–M, *P >*0.05). In contrast, transient optogenetic inhibition of M2-innervated GPe neurons during reward presentation suppressed calcium signals linked to volitional action, auditory feedback, and reward delivery in STN (Fig. 8K, M, *P <*0.05; Fig. 8N, *P <*0.01) but left M1 signals unchanged (Fig. 8F–I, *P >*0.05). These findings indicate that the M2-GPe pathway primarily modulated the auditory feedback response in the GPe, involving the regulation of volitional inhibition. Meanwhile, the inhibition of these neurons also impaired the volitional control task and increased the response time.

## Discussion

### The Volitional Control Task as a Powerful Tool for Basic Scientific Studies of Sensorimotor Integration

The effectiveness of BCI control relies on a well-defined sensorimotor loop involving cognitive functions such as prediction, learning, and multisensory integration [8, 10, 24–29]. The motor BCIs primarily rely on neural circuits associated with movement, without engaging the intricate and extensive muscle system located beneath the brain stem [30–34]. Additionally, BCI models possess the capability to eliminate somatosensory feedback and can also artificially replicate such feedback through direct stimulation of the somatosensory cortex, which can be subsequently integrated into the BCI model [8, 25]. Moreover, the control neurons within the BCIs are specifically tailored by the researcher, thereby establishing a causal link between neuroprosthetic movement and neuronal activity [30]. The aforementioned characteristics render the BCI model an effective tool for investigating sensorimotor integration, with distinct advantages. In the current volitional task, we not only detected various sensory signals across different temporal dimensions but also analyzed neural signals across multiple spatial dimensions (different brain areas). Additionally, transgenic mice with expression of GCaMP6f in various neuronal types facilitate a comprehensive investigation into the mechanisms of sensorimotor integration across distinct neuronal populations, temporal contexts, and spatial dimensions. Furthermore, owing to the causal relationship between neuronal activity and neuroprosthetics in BCI, the combination of optogenetics and chemogenetics facilitates the examination of neuronal population dynamics or oscillation changes by specific sensory inputs on BCI control outcomes. Taken together, these features render the BCI a very useful tool for the investigation of sensorimotor integration.

### GPe Neurons Innervated by M2 are Involved in Sensory Feedback for Volitional Control

Online feedback, which can include auditory, visual, or artificial stimulation, plays a crucial role in facilitating BCI control by transforming the BCI into a bidirectional closed-loop system [10]. Real-time feedback provides BCI users with immediate results regarding their control efforts, enabling them to actively modify their mental activity strategies or select suitable external stimuli to enhance performance stability, accuracy, and timeliness [10, 35, 36]. We demonstrate that GPe neurons receiving the projection from M2 play a crucial role in regulating auditory feedback for volitional inhibition. Notably, the auditory feedback is associated with the control efforts and internal neuronal state. Thus, the inhibition of GPe neurons also impaired volitional control (GO task). Furthermore, GPe neurons receiving projection from M2 also respond to the auditory cue (5000 Hz), but optogenetic manipulation induced suppression of auditory signals at the presentation of the auditory cue failed to influence volitional inhibition. Auditory cues may influence the learning process; however, the mice had already mastered the GO and NO-GO tasks prior to engaging in the volitional stop-signal paradigm. Thus, optogenetic suppression of GPe neurons receiving projection from M2 precisely at the time of auditory feedback likely influenced the volitional inhibition. In addition to the auditory processing, the M2 region also receives projections from the visual and somatosensory cortices. The M2 also exhibits a topographical organization in its reception of sensory projections, with the rostral M2 receiving a greater proportion of somatosensory motor input, while the caudal M2 is predominantly associated with the reception of sensory input. The finding that the M2-GPe projection pathway plays an important role in auditory feedback could open up new opportunities for simulating M2 as biofeedback to enhance the stability and accuracy of volitional inhibition and, therefore, of BCI control.

### The Difference Between BCI Control and Motor Control

Motor BCIs necessitate the acquisition of skills through repetitive practice in order to attain proficient functionality [20–22, 32, 37]. Similar to the control and learning of motor skills, BCIs depend on neural circuits located within the cortical and subcortical basal ganglia regions [38, 39]. Various cortical regions have been implicated in the BCI control [40, 41]. Our previous research has demonstrated that adenosine A2A receptors, which are known to modulate motor skill acquisition, also play a role in the learning processes associated with volitional and hence BCI control [20, 21]. Similarly, at the motivational level, the neural circuits involved in BCI learning and control are comparable to those used in motor learning and control [21]. However, BCIs are directly governed by the modulation of a specific local population of neurons to manipulate external devices [3], whereas motor control involves the brain's regulation of the musculoskeletal system to facilitate motor behaviors. BCI control is executed by the cortex-spinal cord system, resulting in the absence of somatosensory feedback. These distinctions indicate that there are significant disparities between the control mechanisms of BCI and those of motion control systems. Indeed, our recent investigation has demonstrated that various brain regions exhibit contrasting effects on BCI and motor controls [22]. Nonhuman primates perform a two-dimensional, self-initiated, center-out task under BCI control and motor control, demonstrating the presence of distinct neural representations for BCI and manual controls in M1, DLPFC, and Cd [42]. Actually, the absence of the somatosensory feedback represents a clear distinction between the BCI control and motor control. Furthermore, loss of proprioception has a more profound effect on coordinated body movements [43–45]. Consequently, we speculate that the absence of somatosensory feedback may be a critical bottleneck for BCI control to achieve normal motor control. However, it provides a theoretical basis for introducing artificial somatosensory feedback in BCI control by dissecting the neural circuit mechanism of different sensory feedback.

### External Variables Affected BCI Control and Volitional Inhibition

The control of BCIs is affected by a range of internal and external variables [46]. External variables are defined as elements of the environment that primarily exist outside the BCI user and are situated within the BCI system itself. Internal variables are characterized as factors that primarily arise from within the user of the BCI, such as emotion, attention, motivation, and similar elements. The volitional stop-signal task is designed to evaluate the impact of external variables on BCI control, encompassing aspects such as environmental interactions and sensory feedback mechanisms. Our research utilized distinct auditory cues, specifically 5000 Hz and 27000 Hz, to signify interaction with the environment, while a sensory feedback cue at 10,000 Hz was employed to represent sensory feedback. Nonetheless, both external variables can be incorporated via the M2 region [11, 12]. However, environmental interactions occur through the M2-striatal pathway, and sensory feedback is processed through the M2-GPe loop. Finally, these two pathways, integrated with direct and indirect pathways the basal ganglia to BCI control by returning to the M1 region. However, the volitional stop-signal task is primarily designed to assess the ability to inhibit initiated volitional processes in response to environmental changes during BCI control. Volitional inhibition is particularly crucial for the application of BCI in real-world settings. We assume the neural circuits responsible for regulating behavioral inhibition and volitional inhibition may operate through similar mechanisms; however, a key distinction lies in the presence of sensory feedback in volitional control.

## Conclusion

**Fig. 9:**
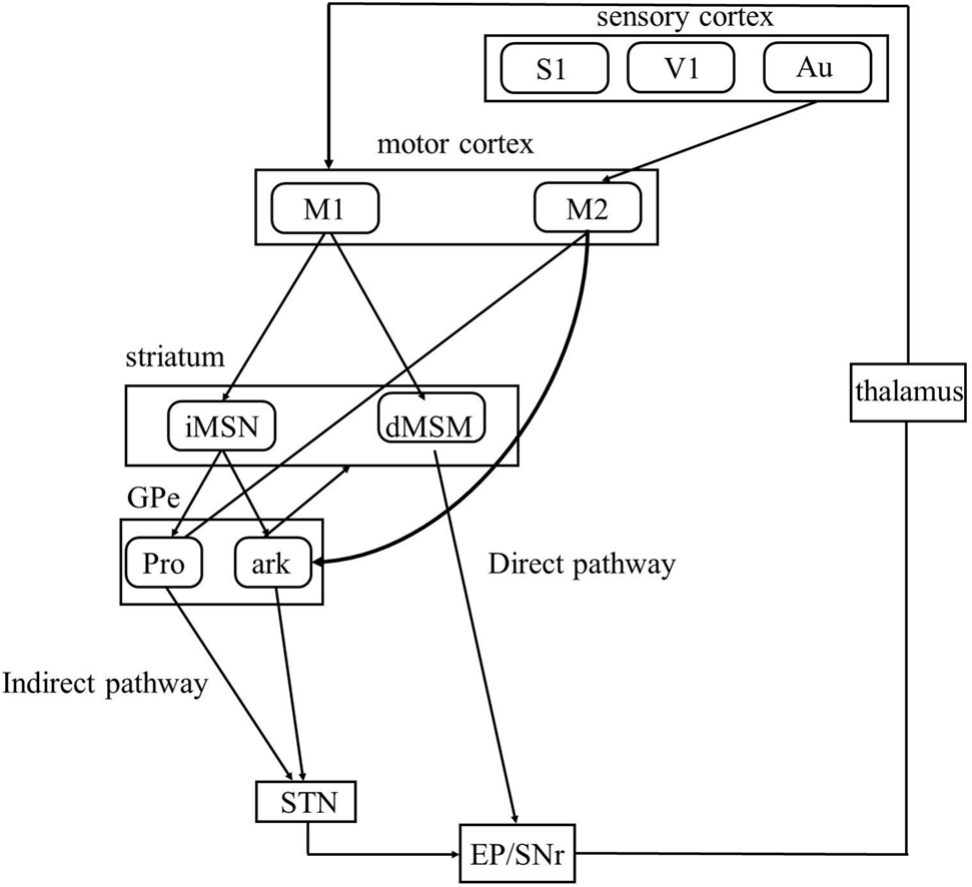
Schematic Diagram of M2-GPe Neurons Involved in Volitional Control. The direct and indirect pathways within the basal ganglia play a critical role in modulating both the acquisition and execution of volitional control. Sensory feedback, including visual, auditory, and somatosensory signals, is integrated via the M2-GPe circuit to the basal ganglia, collectively contributing to the regulation of volitional control.

In summary (Fig. 9), volitional control requires the coordinated activity of the direct and indirect pathways of the basal ganglia to effectively support processes such as learning and executive control. M1 generates volition-initiating neural signals that drive the execution of volitional responses. The GP(e) a component of the indirect pathway that relays striatum inputs to the STN-mediated volitional inhibition and circuit stabilization to support the precision of volitional control. The M2-GPe circuit primarily integrates sensory feedback—including auditory, visual, and tactile inputs—via this pathway, interacting with the direct and indirect pathways of the basal ganglia to modulate volitional control. The M2-GPe circuit contributes to volitional inhibition by influencing the response time of volitional control (GO task).

## Supplementary Information

Below is the link to the electronic supplementary material.

Supplementary file1 (PDF 560 kb)

## Data Availability

Original data and code are available upon request from the corresponding author.
