# Supplementary material for: The Secondary Motor Cortex-External Globus Pallidus Pathway Regulates Auditory Feedback of Volitional Control
Source: Neurosci Bull. 2025 Dec 2;42(6):1218–34. doi: 10.1007/s12264-025-01538-6 (PMC13221500; doi:10.1007/s12264-025-01538-6)
Supplement: Supplementary file 1 — Supplementary file1 (PDF 560 kb) [file 12264_2025_1538_MOESM1_ESM.pdf]

Supplementary Materials

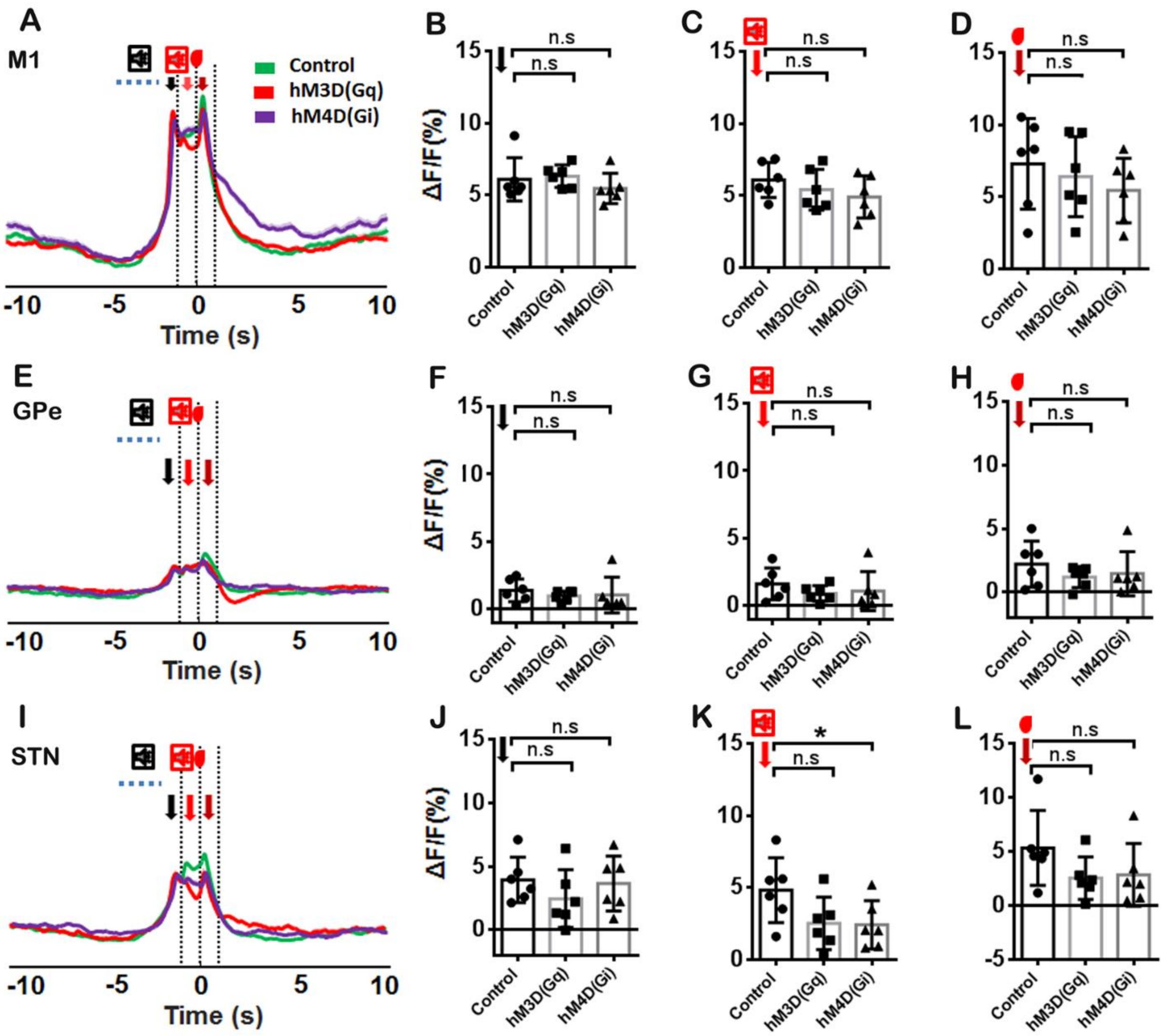

Supplemental Fig. S1 The Analysis of the Calcium Signal before (10 s) and after (10 s) the Reward Delivery for the GO task Without CNO Treatment. A The representation of the calcium signal before (10 s) and after (10 s) the reward delivery for M1. **B** Analysis of peak calcium signal for the volitional signal among control, hM3D(Gq), and hM4D(Gi) groups. **C** Analysis of peak calcium signal for auditory feedback among control, hM3D(Gq), and hM4D(Gi) groups. **D** Analysis of peak calcium signal for reward among control, hM3D(Gq), and hM4D(Gi) groups. **E-H** The same as A-D, but for the GPe. I-L) Same as **A-D**, but for the STN. K: Control vs hM4D(Gi), $P<0.05$.

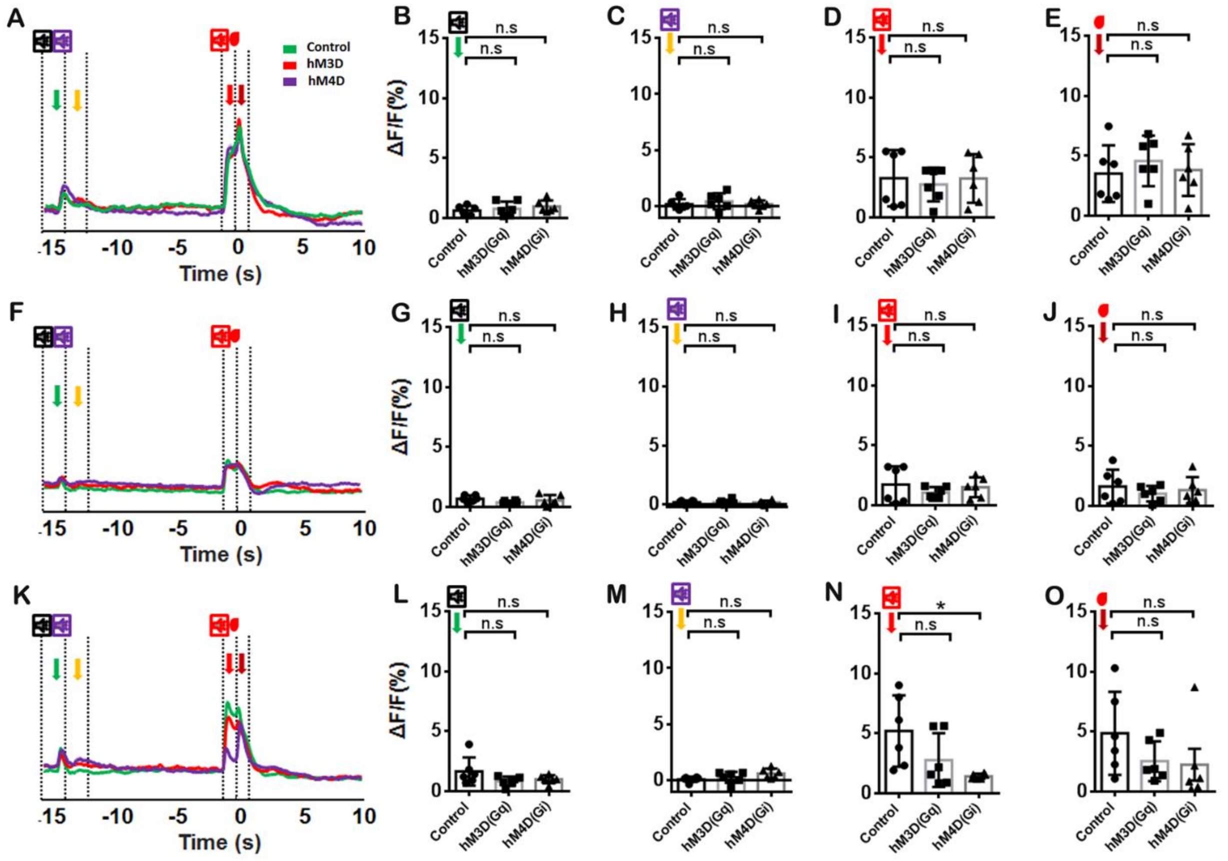

Supplemental Fig. S2 The Analysis of Calcium Signal before (10 s) and after (10 s) the Reward Delivery for the NO-GO Task without CNO Treatment. A The representation of the calcium signal before (10 s) and after (10 s) the reward delivery for M1. **B** Analysis of peak calcium signal for the first auditory cue among control, hM3D(Gq), and hM4D(Gi) groups. **C** Analysis of the peak of the calcium signal for the volitional secondary auditory cue, hM3D(Gq) and hM4D(Gi) group. **D** Analysis of peak calcium signal among control, hM3D(Gq), and hM4D(Gi) groups. **E** Analysis of peak calcium signal for reward among control, hM3D(Gq), and hM4D(Gi) groups. **F**-**G** The same as **A**-**E**, but for the GPe. **K-O** Same as **A**-**D**, but for the STN. N: hM3D(Gq) vs hM4D(Gi), P<0.05. The control group: n = 6; hM4D（Gi）group: n = 6; hM3D（Gq）group: n = 6.

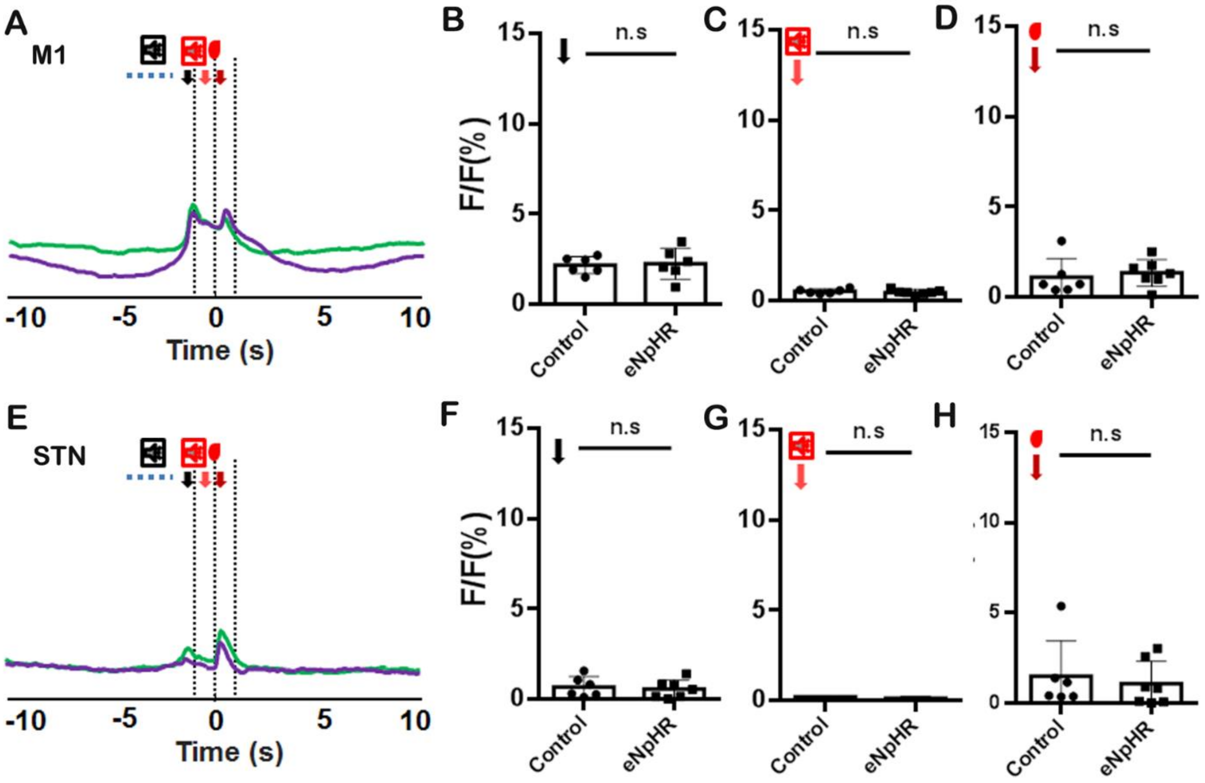

Supplemental Fig. S3 The Analysis of Calcium Signal before (10 s) and after (10 s) the Reward Delivery for the GO Task Without Optogenetic Inhibition. A The representation of the calcium signal before (10 s) and after (10 s) the reward delivery for M1. **B** Analysis of peak calcium signal for the volitional signal between the control and eNpHR groups. **C** Analysis of peak calcium signal for auditory feedback between the control and eNpHR groups. **D** Analysis of peak calcium signal for reward between the control and eNpHR groups. **E-H** Same as **A-D**, but for the STN. The control group: n = 6; eNpHR 3.0 group: n = 7.
